# Acute Myocardial Infarction Mortality Rates and Trends in Romania between 1994 and 2017

**DOI:** 10.3390/ijerph17010285

**Published:** 2019-12-31

**Authors:** Sorin Ioacara, Andreea C. Popescu, Joseph Tenenbaum, Doina R. Dimulescu, Mihaela R. Popescu, Anca Sirbu, Simona Fica

**Affiliations:** 1 Carol Davila University of Medicine and Pharmacy, 050474 Bucharest, Romania; drsorin@yahoo.com (S.I.); andreea.catarina.popescu@gmail.com (A.C.P.); doina.dimulescu@gmail.com (D.R.D.); anca.sirbu@umfcd.ro (A.S.); simona.fica@umfcd.ro (S.F.); 2 Endocrinology Department, Elias University Emergency Hospital, 011461 Bucharest, Romania; 3 Cardiology Department, Elias University Emergency Hospital, 011461 Bucharest, Romania; 4 Cardiology Department, Columbia University, New York, NY 10027, USA; jt7@cumc.columbia.edu

**Keywords:** mortality trend, myocardial infarction, life expectancy, healthcare programs

## Abstract

*Introduction:* The current study aimed to assess recent acute myocardial infarction (AMI) mortality rates and trends in Romania between 1994 and 2017. This dataset is a necessity in the context of the current improvement of emergency protocols, medical addressability, and modernization of hospital infrastructure. *Materials and Methods:* The study is a retrospective analysis of an anonymized mortality database containing all deaths registered in Romania during 1994–2017. AMI crude mortality rates (CMR) and age-standardized mortality rates (ASMR) were calculated using the European Standard Population. Poisson regression was used for calculating the annual percentage change (APC) in mortality, subsequently used to make mortality predictions through the year 2030. *Results:* There were 197,152 AMI deaths in women (39.3% of total AMI), and 304,644 (60.7%) in men. Mortality rates were higher in men as compared with women for the entire time covered by the study. Based on the 1994–2017 ASMR dynamics, predictions for the year 2030 showed an overall AMI ASMR of 70.9 (95% CI 69.9–71.9), with gender analysis showing 46.8 (95% CI 45.8–47.9) in women and 104.1 (95% CI 102.3–105.8) in men. *Conclusion:* Acute myocardial infarction age-standardized mortality rates decreased significantly in Romania between 1994 and 2017 in close correlation to the implementation of national healthcare programs.

## 1. Introduction

According to the Global Burden of Disease Study 2017, cardiovascular diseases were responsible for 31.8% of all deaths worldwide [1]. The global age-standardized death rate (ASDR) was 233.1 per 100,000 persons, declining 10.3% during the last decade [1]. Approximately half of all cardiovascular deaths were due to ischemic heart disease, which also declined by 9.7% during 2007–2017 [1]. However, significant geographical variations were reported, with Romania showing some interesting particularities. First, cardiovascular diseases were responsible for a significantly higher percentage of total deaths (55.5%). Second, the death rates for cardiovascular diseases were roughly three times higher in Romania compared with the rest of the world. And third, the cardiovascular death rate (per 100,000) increased during the last decade from 681 (CI 95% 677–685) in 2007 to 745 (CI 95% 716–774) in 2017, as opposed to the general tendency towards a reduction [1].

Acute myocardial infarction (AMI) is the leading cause of death among ischemic heart diseases. AMI mortality data was not available for analysis in the Global Burden of Disease Study 2017. All previously published data on AMI mortality rates and trends in Romania were based on various samples of AMI cases admitted in the hospital, especially for angioplasty procedures [2,3]. AMI mortality is significantly higher before reaching the hospital, with a second drop in mortality rate after the initial 2 h from hospital admission. Higher-ranked hospitals, with clear AMI protocols, including access to revascularization procedures, tend to have better results in terms of AMI mortality. In this context, the current study aimed to assess AMI mortality at the national level, including all individual AMI deaths registered in Romania, starting with 1994 and through to 2017.

## 2. Materials and Methods

The present study is a retrospective analysis of an anonymized mortality database obtained from the National Institute of Statistics (Eurostat microdata). It comprises all deaths registered in Romania during 1994–2017, using the information from the death certificate. Exposure was also available as mean estimates of the resident population on July 1st, for each year of interest [4].

All death-related data was obtained from the death certificate. Gender, date of birth, year of death, cause of death, and place of death were accessible in this study. During 1994–1999, causes of death were encoded using a local system related to the International Statistical Classification of Diseases and Related Health Problems (ICD) 9th revision. The ICD 10th revision was used starting with the year 2000 (I21–I23). Around 99% of the cases used in this study were coded as I21 (acute myocardial infarction). The date July 1st was used for each time of death as the exact day and month of death were not available (Eurostat anonymized microdata). This date was chosen for minimizing the error in age at death calculation. Also, the matching exposed general population was counted as of July 1st each year.

Acute myocardial infarction (AMI) crude mortality rates (CMR) and age-standardized mortality rates (ASMR) were calculated using the European Standard Population [5]. We used five years age groups, starting at birth, with the last group, including all deaths, starting at age 85 years and above. The last age band was constructed in corroboration with the accessibility of the general population data. Some discrepancies between the ASMRs reported in this study and other officially declared data might be explained by the adjustment used for deaths occurring after the age of 85 years, as the last age band in some official data might be ≥95 years.

Another minor source of variations could reside in error in age at death measurement (see above). However, due to the relatively large number of cases analyzed here, the positive and negative misclassified cases should effectively cancel each other out. CMRs for age groups less than 40 years were not included in the data tables (see Results) due to the small number of cases. They were, however, used in the overall and age-standardized mortality analysis.

Poisson regression was used for calculating the annual percentage change (APC) in mortality. Analysis of trend used the APC recurrently applied to the last available ASMR (year 2017). The aim was to make mortality predictions through to the year 2030. Mortality life table analysis for the year 2017 was used to evaluate the gain in life expectancy at birth that could be obtained by completely removing the AMI-related mortality. Statistical analysis was performed using Stata version 13 (StataCorp LLC, College Station, TX, USA).

The local ethics committee approved the study. Informed consent was not obtained because this was a retrospective, anonymized database-driven study. Written permission for data usage was obtained from the Eurostat/National Institute of Statistics.

## 3. Results

**Figure 1:**
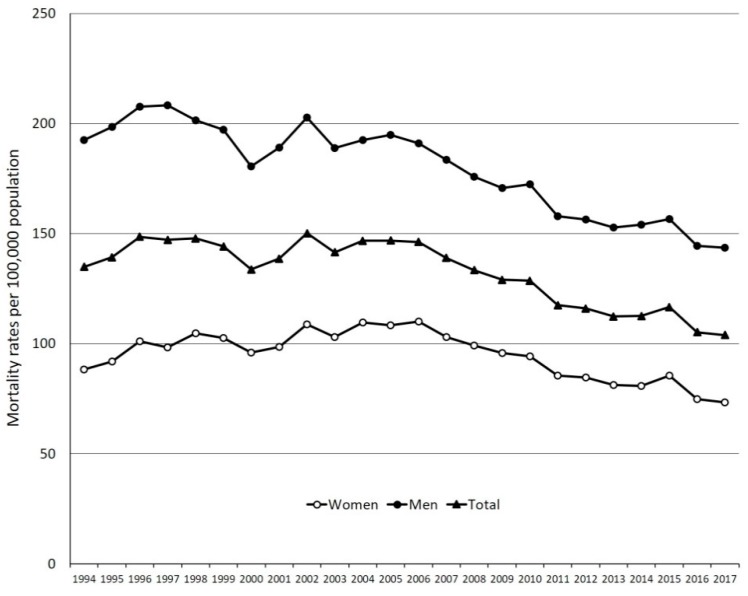
Acute myocardial infarction age-standardized mortality rates in Romania (1994–2017).

AMI was registered as the underlying cause of death for 501,796 cases (8% of total deaths) during 1994–2017. There were 197,152 AMI deaths in women (39.3% of total AMI) and 304,644 (60.7%) in men. The corresponding alive general population showed a linear decrease from 22.7 million in 1994 to 19.6 million in 2017. The overall AMI CMR per 100,000 person-years was 98.5 (75.5 for women and 122.5 for men). All CMRs increased with age in both women (Table A1) and men (Table A2). There was a significant decrease in AMI overall CMR and age-standardized mortality rates (ASMR), as summarized in Figure 1 and Table A3 and Table A4 (see Appendix A). This decrease was highest in the 40–44 age group and decreased in amplitude with age (Table A1 and Table A2).

Life table analysis for the year 2017 showed that complete removal of AMI mortality would result in 0.78 years gain in life expectancy at birth (0.52 years in women and 0.96 years in men).

There was an initial significant increase in AMI ASMR per 100,000 person-years from 134.9 (95% CI 134.3–135.6) in 1994 to 146.2 (95% CI 145.6–146.8, *p* < 0.001) in 2006. However, this increase was not steady but marked by high fluctuations (see Figure 1). This initial increase in ASMR was followed by a significant decrease to 103.9 (95% CI 103.5–104.4, *p* < 0.001) in 2017, with an APC of 2.9% per year (95% CI 2.8–3.0, *p* < 0.001). This decrease in ASMR was steeper in women (APC 3.4%, CI 95% 3.2–3.6) as compared with men (APC 2.4%, CI 95% 2.3–2.6). Mortality rates were higher in men as compared with women for the entire time covered by the study (Figure 1). Based on the 1994–2017 ASMR dynamics, predictions for the year 2030 showed an overall AMI ASMR of 70.9 (95% CI 69.9–71.9), with gender analysis showing 46.8 (95% CI 45.8–47.9) in women and 104.1 (95% CI 102.3–105.8) in men.

Most patients died of AMI at home (*n* = 335,521, 66.9%), followed by in hospital (*n* = 121,411, 24.2%) and elsewhere (*n* = 44,950, 0.9%). Deaths from other causes than AMI were more likely to happen at home (70.5%, *p* < 0.001) and less likely in the hospital (22.0%, *p* < 0.001). Deaths registered at home significantly decreased from 69.6% (95% CI 69.0–70.3) in 1994 to 67.9% in 2006 (95% CI 67.3–68.5, *p* < 0.001 vs. 1994) and 59.8% in 2017 (95% CI 59.1–60.5, *p* < 0.001 vs. 2006). There was also a significant increase in the proportion of AMI deaths registered in the hospital, from 20.3% (95% CI 19.7–20.9) in 1994 to 22.8% (95% CI 22.2–23.3, *p* < 0.001 vs. 1994) in 2006 and 30.4% (95% CI 29.8–31.1, *p* < 0.001 vs. 2006) in 2017.

The mean age at AMI death increased significantly in both women (70.7 ± 12.5 years in 1994 vs. 78.1 ± 11.1 years in 2017, *p* < 0.001) and men (63.0 ± 13.3 years in 1994 vs. 70.0 ± 12.9 years in 2017, *p* < 0.001). This trend was accompanied by a significant increase in the mean age at death from causes other than AMI in both women (70.9 ± 18.9 years in 1994 vs. 77.7 ± 14.0 years in 2017, *p* < 0.001) and men (63.9 ± 20.0 years in 1994 vs. 70.7 ± 15.6 years in 2017, *p* < 0.001).

## 4. Discussion

Acute myocardial infarction age-standardized mortality rates decreased significantly in Romania during 1994–2017. However, this was not a linear decrease, with data showing an initial increase in mortality rates during 1994–2006, followed by a steady decline until 2017. This fact could be partly explained by new healthcare national initiatives aligned with the European guidelines on the matter, better addressability to emergency units, and better effectiveness of the ambulance services. Providing a socio-economic context might give some clues about the dynamic of AMI mortality reported in the current study. The transition towards the western European lifestyle started in 1989, with a significant step forward in 2007, represented by the country’s admission to the European Union. Major healthcare reforms took place immediately before and after. This pivotal moment led to significantly increased access to outpatient health care. The low complexity cases were offloaded from the hospitals and allowed for better accessibility of complex cases (including AMI) to the much-needed hospital care. This might have at least partially contributed towards the shift from home to hospital location of AMI death registration. This shift was already apparent by 2006, but significantly amplified thereafter. The National Program for Primary Angioplasty in ST-segment Elevation Myocardial Infarction started in August 2010, and a national media campaign for raising public awareness of myocardial infarction symptoms was conducted during 2015–2016. Furthermore, there was a significant development in interventional cardiology, such as novel stents and potent antiplatelet agents, and enforcement of evidence-based medicine. The improved hospital access and care for AMI patients might have also contributed towards the recent decline in AMI mortality. A report from the Romanian ST-Elevation Myocardial Infarction registry (RO-STEMI) analyzing data from 15,076 patients with ST-elevation AMI showed that 77% of them received a percutaneous coronary intervention, with significantly better in-hospital mortality rates as compared with conventional treatment (4.1% vs. 15.7%) [6]. To conclude, cigarette smoking was forbidden in public places in Romania, starting in March 2016. The impact of this measure on the AMI mortality trend is more likely minor in the current study, but might have implications for future studies. As per the data published in 2009 from the Romanian Registry for ST-segment Elevation Myocardial Infarction, only a very small proportion of the patients underwent primary percutaneous coronary intervention (PCI) (1.9%) [2]. The data for this study was collected starting with 2000 and ending in 2007. Thrombolysis was used much more than PCI, in about a third of patients.

As PCI was proven to be more efficient than thrombolysis, the PCI utilization in Europe was approximated to have reached 50% by 2009 [7]. In a paper published in 2010, regarding the situation in Europe between 2007 and 2008, Romania still predominantly used thrombolysis, with only 5% PCI use [8]. By 2010, the PCI rate was still 25%, but after the introduction of the national PCI program, it reached 49.32% in 2011 [9]. However, the data indicates an increase in PCI utilization from 1.9% to 25% between 2000 and 2010. This escalation was somewhat linear, explaining why the mortality rate started to decline at about half the interval, specifically in 2006. Moreover, improvement in prehospital care also contributed to this decline [10].

On the other hand, the initial increase in mortality may be due to a combination of factors. One factor is the already mentioned switch to an unhealthy, fast-food laden, and more sedentary lifestyle after the 1989 fall of the communist regime. Also, another key element is the publishing of the first universal definition of the myocardial infarction in 2007. This guideline unifies the diagnostic criteria, making over diagnosing of AMI less probable, thus contributing to the decline observed after 2006. Moreover, better diagnosis (use of troponin, creatine kinase, creatine kinase — MB) aided in the process of recording a correct diagnosis.

Gender analysis showed that only women experienced this initial increase, with men displaying a relatively steady AMI ASMR. The decline in ASMR in the last decade was apparent in both men and women. AMI mortality rates were higher in men as compared with women for the entire study period. They increased with age in both men and women.

A previous report showed a significant decrease of around 25% in ischemic heart disease mortality in Romania during 2002–2012 [11]. A general reduction in AMI mortality was also reported in other studies [1,12,13]. In a recent report from the American Heart Association, the incidence and mortality from ischemic heart disease was reported to be approximately 10–20 years lagging in women as compared with men [14]. Hypertension, dyslipidemia [15], smoking, obesity, and diabetes [16,17] are major cardiovascular risk factors, commonly found among adult Romanian population. Hypertension prevalence in Romania reported by the SEPHAR III study was 45.1%, increasing from 40% in the previous report [18,19]. This increase in incidence was apparently contrasting with the reported decrease in AMI mortality during the same period. The explanation may reside in the significant rise in the prevalence of hypertensive patients receiving treatment (72.2 vs. 59.2%), achieving the treatment target (30.8 vs. 25%), and aware of having hypertension (80.9 vs. 69.6%) [18]. Romania is experiencing a relatively high prevalence of obesity (31.9%), increased waist circumference (73.9%), metabolic syndrome (38.5%), and diabetes (11.6%) [20,21]. As Romanian patients with diabetes live longer, an increased burden on cardiovascular mortality is to be expected, opposing the general tendency of cardiovascular mortality decline registered in recent years [22,23,24].

The strength of the current study resides in full national coverage of all AMI deaths over an extensive period, with an estimated rate of case capture very close to 100%. Also, this study is part of a bigger body of work regarding cardiovascular death. The already published data on stroke mortality shows similar trends [25].

Although ischemic heart disease mortality data can be obtained from other publicly available databases, AMI-related data cannot. Also, access is generally open to gender and age group statistics. Case by case access to mortality data is not currently available to the public. An acknowledged weakness of the study comes from the inherent drawbacks of using death certificates as the source of mortality data, especially in the context of a meagre autopsy rate. Although miscoding the cause of death is a recognized source of error, it generally presents more like a systematic than a random error. Therefore, its impact on trend analysis, as done in this study, could be considered acceptable. Some recent AMI public awareness campaigns might have increased the likelihood of AMI coding on the death certificates, contributing to an underestimating of the actual decline in AMI mortality in recent years. Therefore, the reported reductions in AMI mortality could be considered as conservative figures. The socio-economical context provided here is strictly informational, with no definitive data to support any causality.

## 5. Conclusions

Age-standardized acute myocardial infarction mortality in Romania increased during 1994–2006, followed by a significant decrease until 2017. Mortality rates were higher in men compared with women and increased with age in both genders. Conservative predictions for 2030 showed a continued decline in AMI mortality rates. The possible explanation for these trends resides in the western, sedentary lifestyle, bad eating habits, which were gradually buffered by national health awareness initiatives, better addressability to healthcare professionals, and better healthcare national programs. Still, by the middle of the last decade (around 2006) the proficiency of the healthcare system had not yet reached western European standards.

## Appendix A

**Table A1:** Acute myocardial infarction crude mortality rates in women, by age and calendar year.

Year	Women Crude Mortality Rates Per 100,000 Person-Years, by Age Group (Years)									
	40–44	45–49	50–54	55–59	60–64	65–69	70–74	75–79	80–84	≥85
1994	16.8	30.3	47.5	70.5	110.2	170.3	256.9	388.4	477.3	794.8
1995	16.6	27.8	48.1	70.7	116.9	189.9	268.1	382.4	520.0	828.6
1996	18.1	32.5	54.8	76.2	126.8	181.9	267.5	411.2	618.7	984.5
1997	17.1	32.5	48.7	81.0	120.3	200.0	266.4	383.5	610.7	923.3
1998	17.3	31.5	47.2	82.3	125.0	185.8	293.7	443.6	631.8	1029.8
1999	14.5	24.1	46.9	73.6	113.5	187.0	281.4	444.0	652.0	1023.7
2000	14.2	22.5	38.1	71.7	110.2	165.3	260.9	398.7	578.5	1050.2
2001	15.7	26.7	47.0	69.3	110.6	176.6	267.7	406.0	615.3	1027.9
2002	12.9	26.9	44.3	76.4	119.5	184.2	290.4	436.8	697.8	1217.2
2003	15.8	25.1	38.4	76.9	109.7	170.5	284.7	421.7	619.6	1172.8
2004	14.9	24.4	38.2	65.4	110.4	181.3	294.6	447.6	660.6	1344.6
2005	11.5	21.4	41.4	64.8	115.9	172.0	283.6	468.4	706.8	1251.2
2006	10.6	21.0	38.4	64.5	101.8	169.6	284.7	439.0	661.2	1251.3
2007	10.5	21.3	31.3	67.0	97.3	163.9	257.5	449.5	676.1	1249.2
2008	8.4	20.1	31.8	57.0	93.8	151.1	262.6	426.4	654.1	1210.7
2009	9.0	18.3	30.5	57.5	96.4	146.3	245.9	407.3	608.2	1195.1
2010	10.2	16.4	30.1	44.9	83.6	141.4	246.1	396.6	633.5	1199.9
2011	8.6	16.7	24.4	45.1	77.3	122.7	217.5	361.5	592.7	1083.1
2012	6.0	14.4	24.3	42.2	73.6	121.6	203.8	366.3	593.0	1096.3
2013	7.7	9.0	27.2	42.7	73.5	118.2	207.5	337.5	568.5	1040.4
2014	5.6	13.0	23.7	41.5	70.7	114.6	197.9	327.1	595.0	1049.7
2015	7.4	12.0	22.9	43.7	72.0	119.3	203.3	345.4	598.8	1179.8
2016	6.0	10.3	18.8	39.8	63.8	111.3	181.4	311.9	540.6	977.1
2017	6.1	9.6	24.2	37.7	66.6	107.1	173.4	297.9	537.0	948.7

**Table A2:** Acute myocardial infarction crude mortality rates in men, by age and calendar year.

Year	Men Crude Mortality Rates Per 100,000 Person-Years, by Age Group (Years)									
	40–44	45–49	50–54	55–59	60–64	65–69	70–74	75–79	80–84	≥85
1994	91.5	134.5	214.9	261.0	340.0	429.4	532.3	595.3	774.3	1012.3
1995	88.6	136.0	211.1	272.9	342.0	427.7	541.3	661.7	811.4	1085.3
1996	87.0	150.1	214.7	279.7	359.1	433.0	556.6	699.2	882.0	1170.7
1997	94.7	141.9	207.4	277.0	364.5	455.9	566.0	707.2	828.1	1174.8
1998	80.1	129.8	196.4	268.2	335.7	430.7	547.2	691.3	935.0	1152.4
1999	70.8	123.2	177.1	233.8	321.5	424.9	540.5	675.0	898.0	1319.9
2000	68.3	108.4	163.8	232.8	310.2	384.9	496.1	649.1	764.2	1137.9
2001	61.8	114.7	165.8	245.2	332.4	405.2	521.9	676.2	767.2	1253.3
2002	68.5	118.8	175.5	238.8	320.6	425.0	554.1	731.1	905.2	1453.3
2003	64.1	110.4	170.5	245.3	336.0	424.7	545.2	685.6	875.8	1030.6
2004	63.8	105.3	171.8	240.0	319.7	435.9	534.6	718.6	948.0	1105.6
2005	52.5	110.5	159.3	232.8	315.6	435.6	549.1	722.5	963.4	1252.9
2006	54.8	98.0	161.5	225.9	318.8	411.0	521.4	720.0	1003.0	1199.8
2007	46.3	94.3	154.7	221.4	312.5	384.9	511.3	719.1	962.9	1112.4
2008	40.2	87.9	157.6	217.3	286.5	369.2	514.4	673.3	863.4	1099.4
2009	39.7	85.6	142.1	201.0	284.9	365.1	499.5	650.1	876.7	1062.3
2010	39.3	84.5	132.3	198.4	273.2	369.7	483.0	652.5	959.9	1142.9
2011	35.7	69.4	127.2	184.8	265.2	316.0	458.6	597.1	876.5	1031.0
2012	36.7	67.2	116.8	179.5	241.5	324.5	400.6	555.6	836.3	1291.0
2013	32.0	61.9	108.1	170.3	237.6	315.2	413.3	558.7	801.0	1249.1
2014	28.4	59.5	110.1	173.2	236.4	309.3	411.6	576.3	806.9	1293.6
2015	30.5	55.0	103.3	162.9	245.3	315.2	427.8	617.4	840.6	1286.2
2016	28.7	52.1	104.6	156.0	218.1	304.1	402.1	554.8	760.9	1160.2
2017	25.5	54.5	104.2	155.4	228.5	314.0	396.7	523.78	736.39	1156.8

**Table A3:** Acute myocardial infarction crude mortality rates in Romania (1994–2017).

Year	Women		Men		Total	
	N ^a^	CMR ^b^	Na	CMR ^b^	N ^a^	CMR ^b^
1994	6684	57.75	13,216	118.5	19,900	87.5
1995	7116	61.6	13,555	121.9	20,671	91.1
1996	7737	67.1	14,108	127.3	21,845	96.6
1997	7730	67.2	14,313	129.6	22,043	97.8
1998	8211	71.5	13,651	124.0	21,862	97.2
1999	8038	70.1	13,153	119.7	21,191	94.4
2000	7600	66.3	12,365	112.7	19,965	89.0
2001	8025	70.0	13,022	118.9	21,047	93.9
2002	8670	78.0	13,482	127.6	22,152	102.2
2003	8370	75.3	13,336	126.8	21,706	100.4
2004	8831	79.9	13,515	129.1	22,346	103.8
2005	9008	82.0	13,618	130.9	22,626	105.8
2006	9161	83.9	13,461	130.1	22,622	106.4
2007	8871	81.8	13,131	127.7	22,002	104.1
2008	8760	82.4	12,782	127.7	21,542	104.4
2009	8621	81.9	12,499	126.1	21,120	103.3
2010	8675	83.0	12,638	128.5	21,313	105.0
2011	8111	78.5	11,770	120.2	19,881	98.8
2012	8167	78.9	11,353	116.5	19,520	97.1
2013	8094	79.1	11,116	113.9	19,210	96.1
2014	8207	80.6	11,309	116.2	19,516	98.0
2015	8813	86.9	11,604	119.9	20,417	103.0
2016	7877	78.2	10,815	112.3	18,692	94.9
2017	7775	77.4	10,832	112.8	18,607	94.7

^a^ Number of cases; ^b^ crude mortality rate per 100,000 person-years.

**Table A4:** Acute myocardial infarction age-standardized mortality rates in Romania (1994–2017).

Year	Women		Men		Total	
	ASMR ^a^	95% CI	ASMR ^a^	95% CI	ASMR ^a^	95% CI
1994	88.3	87.6–89.0	192.5	191.4–193.7	134.9	134.3–135.6
1995	91.9	91.2–92.6	198.5	197.4–199.7	139.3	138.7–140.0
1996	101.1	100.2–101.7	207.7	206.5–208.9	148.5	147.8–149.2
1997	98.4	97.7–99.1	208.2	207.0–209.4	147.2	146.5–147.8
1998	104.8	104.1–105.5	201.4	200.3–202.6	147.8	147.1–148.4
1999	102.5	101.7–103.2	197.2	196.1–198.4	144.2	143.6–144.8
2000	95.9	95.2–96.7	180.6	179.5–181.7	133.7	133.1–134.3
2001	98.6	97.9–99.3	189.1	188.0–190.2	138.6	138.0–139.3
2002	108.7	107.9–109.4	202.8	201.7–204.0	150.1	149.4–150.7
2003	103.0	102.3–103.8	188.9	187.9–190.0	141.5	140.9–142.1
2004	109.6	108.9–110.4	192.4	191.3–193.4	146.7	146.1–147.4
2005	108.3	107.6–109.1	194.9	193.8–196.0	146.8	146.2–147.4
2006	110.1	109.4–110.9	190.9	189.8–192.0	146.2	145.6–146.8
2007	103.1	102.4–103.8	183.5	182.5–184.5	138.9	138.3–139.5
2008	99.2	98.6–99.9	175.8	174.8–176.8	133.4	132.8–134.0
2009	95.8	95.1–96.4	170.7	169.7–171.6	129.0	128.5–129.6
2010	94.2	93.5–94.8	172.4	171.4–173.3	128.6	128.1–129.2
2011	85.4	84.8–86.0	158.0	157.0–158.9	117.5	117.0–118.0
2012	84.6	84.0–85.1	156.4	155.4–157.3	116.0	115.5–116.5
2013	81.3	80.7–81.8	152.8	151.9–153.7	112.4	111.9–112.9
2014	80.7	80.1–81.2	154.1	153.2–155.0	112.6	112.1–113.1
2015	85.5	84.9–86.0	156.7	155.8–157.6	116.6	116.1–117.1
2016	74.8	74.3–75.3	144.5	143.6–145.4	105.2	104.7–105.6
2017	73.3	72.8–73.8	143.5	142.7–144.4	103.9	103.5–104.4

^a^ Age Standardized Mortality Rate.
